# Postural changes in retinal vascular parameters and risk of diabetic retinopathy progression in type 2 diabetes mellitus: a pilot study

**DOI:** 10.1186/s40662-025-00471-z

**Published:** 2026-01-06

**Authors:** Truong X. Nguyen, Yu Meng Wang, Anni Ling, Chunwen Zheng, Kenny H. W. Lai, Ziqi Tang, John H. K. Liu, Carol Y. Cheung

**Affiliations:** 1 Department of Ophthalmology and Visual Sciences, The Chinese University of Hong Kong, 4/F Hong Kong Eye Hospital, 147K Argyle Street, Jiulong, Hong Kong Special Administrative Region, China; 2 C-MER International Eye Care Group, Hong Kong Special Administrative Region, China; 3 Viterbi Family Department of Ophthalmology, Shiley Eye Institute, University of California, San Diego, La Jolla, CA USA

**Keywords:** Diabetic retinopathy, Diabetes mellitus, Retinal vessels, Smartphone fundus imaging, Postural change

## Abstract

**Background:**

To investigate whether postural changes in retinal vascular parameters, measured using smartphone-based imaging, differ between healthy individuals and patients with diabetes mellitus (DM), and whether these changes independently predict diabetic retinopathy (DR) progression over a 5-year period.

**Methods:**

Retinal images were captured using a smartphone with a clip-on adapter lens in sitting and supine positions. Vascular parameters (caliber, fractal dimension, tortuosity, branching) were quantified using the Singapore I Vessel Assessment software. Cross-sectional analyses compared postural-induced vascular responses between 38 healthy controls and 49 DM participants. DM participants were followed for 5 years, with DR progression defined as ≥ 2-step increase in severity on the Early Treatment Diabetic Retinopathy Study scale. Cox proportional hazards models evaluated associations between baseline postural changes and DR progression.

**Results:**

Healthy controls exhibited significant arteriolar and venular constriction upon moving from sitting to supine (*P* < 0.05). In contrast, participants with DM showed diminished or paradoxical responses that did not reach statistical significance. Significant linear trends were observed for arteriolar caliber, fractal dimension, and simple tortuosity across healthy controls, DM with and without DR (all *P*-trend < 0.05). Greater arteriolar tortuosity was associated with a 2.41-fold higher risk of DR progression (HR = 2.41, 95% CI: 1.37–4.23; *P* = 0.002), while wider venular branching angles correlated with a 45% lower risk (HR = 0.55, 95% CI: 0.35–0.87; *P* = 0.011). Adding these parameters improved predictive discrimination beyond established factors (arteriolar tortuosity: C-statistic 0.630–0.740; venular branching angle: 0.630–0.683; *P* < 0.05).

**Conclusions:**

Smartphone-based imaging of retinal vascular responses to postural changes provides additional prognostic value for DR progression, potentially enhancing early risk stratification and proactive management.

**Supplementary Information:**

The online version contains supplementary material available at 10.1186/s40662-025-00471-z.

## Background

Diabetic retinopathy (DR), a leading global cause of preventable blindness, represents a common microvascular complication of diabetes mellitus (DM) [1]. Traditional risk factors do not fully explain the risk of an individual DR risk [2–4], while current annual fundus photography screening may be unnecessarily frequent for many without vision‑threatening disease [5–9]. Additional biomarkers are needed to improve risk stratification, enable timely referral, and optimize screening intervals.

The retinal vasculature represents a unique window to observe microvascular changes in DM, as it is the only location where blood vessels can be directly visualized non-invasively. Emerging evidence indicates that retinal vessels show subtle functional abnormalities before clinical retinopathy becomes apparent [10–12]. Of particular importance is the intrinsic autoregulatory capacity of these vessels, a mechanism that maintains stable blood flow despite fluctuations in perfusion pressure, which appears to be compromised early in DM [13–15]. For example, under normal physiological conditions, transitioning from the sitting to supine position increases ocular perfusion pressure, triggering a compensatory constriction of retinal arterioles through a myogenic response [16–18]. In patients with DM, hyperglycemia-induced endothelial dysfunction attenuates this adaptive response, a phenomenon that has been associated with increased risk of DR progression in type 1 diabetic individuals [17]. Longitudinal studies suggest that this “defective myogenic response” may serve as an early predictive biomarker of DR, potentially revealing subclinical microvascular compromise before clinical signs become detectable [18].

Despite its promising clinical implication, the assessment of these postural vascular changes has been limited by practical constraints of conventional desktop fundus cameras, which are bulky, expensive, and poorly suited for imaging patients in the supine position. Developments in smartphone-based retinal imaging present a feasible alternative by offering portable, cost-effective devices capable of capturing high-resolution fundus images across different body positions [19, 20]. This innovation is particularly beneficial for rural or resource-limited settings, where specialized ophthalmic equipment and frequent laboratory tests for glycemic monitoring may be unavailable. Paralleling these hardware developments, advanced algorithms for retinal image analysis can now quantify multiple geometric parameters, such as vessel caliber, fractal dimension, tortuosity and branching patterns, which have demonstrated significant prognostic value for DR progression when applied to traditional desk-top fundus photography [21–24]. However, a significant knowledge gap remains regarding which parameters of these postural vascular changes captured via smartphone imaging provide superior prognostic value compared with established risk factors in predicting DR progression.

This study first compares retinal vascular parameters between patients with DM and healthy controls during postural changes using smartphone-based imaging, then determines whether these dynamic vascular responses independently predict subsequent DR progression. We hypothesize that measuring will reveal subclinical vascular dysfunction earlier and provide additional prognostic information beyond established risk factors alone.

## Methods

### Study design and participants

This study included two analyses: (1) a cross-sectional, case-control analysis comparing retinal vascular parameters among individuals with DM, with and without DR, and age-matched healthy controls; and (2) a prospective longitudinal analysis evaluating whether baseline postural changes in retinal vascular parameters could independently predict DR progression with an average 5-year follow-up period.

Participants were consecutively recruited from the Chinese University of Hong Kong Eye Center between August 2018 and August 2019. All participants attended their second visit 6 months after the baseline examination. Participants without DR or only mild non-proliferative diabetic retinopathy (NPDR) were followed annually thereafter, whereas those with moderate or severe NPDR were followed every 6 months. The study was approved by the Kowloon Central/Kowloon East Research Ethics Committee (KC/KE‑15‑0137/ER‑3) and conducted in accordance with the Declaration of Helsinki. Written informed consent was obtained from all participants prior to enrolment.

Inclusion criteria were (1) age older than 18 years; (2) confirmed diagnosis of type 2 DM based on standard criteria (fasting plasma glucose ≥ 7.0 mmol/L, 2‑h plasma glucose ≥ 11.1 mmol/L during an oral glucose tolerance test, or HbA1c ≥ 6.5%) [25]; and (3) eyes classified as having no DR or NPDR at baseline. Exclusion criteria were (1) eyes presenting with proliferative DR or diabetic macular edema at baseline; (2) history of panretinal photocoagulation, focal or laser treatment intravitreal anti-vascular endothelial growth factor injections; (3) ocular surgery (including cataract surgery) within 6 months prior to recruitment; (4) ungradable fundus photographs; and (5) concomitant ocular disorders besides DR (e.g., epiretinal membrane, any form of glaucoma, retinal vein occlusion, or neovascular age-related macular degeneration) at baseline or during follow-ups. Both eyes were enrolled if eligible.

### Smartphone retinal vascular imaging

Retinal images were captured using a smartphone equipped with a specialized clip-on adapter lens (OphthoLens, RainsOptics Ltd, Hong Kong) positioned over the device’s camera and flashlight. For each participant, imaging was performed before and after pharmacological pupil dilation, with sequential assessment in sitting and supine positions. Position changes were carefully guided by a trained technician to ensure standardization (Supplementary Figure 1).

Prior to image acquisition, participants rested for 10 min in the sitting position, followed by an additional 10-min rest period after transitioning to the supine position to minimize acute hemodynamic fluctuations. Brachial artery blood pressure (model Avant 2120; Nonin Medical, Inc., Plymouth, MN, USA) and intraocular pressure (Icare PRO tonometer, Tiolat Oy, Helsinki, Finland) were measured in the sitting and supine positions. The smartphone with clip-on adapter lens, blood pressure monitor and intraocular pressure tonometer were held stationary while acquiring data by trained technicians.

All images captured via smartphone underwent a standardized quality assessment that evaluated image clarity, field of view, and resolution. Images failing to meet predetermined quality standards were excluded or recaptured. To evaluate measurement reproducibility, a subset of 30 participants (15 healthy controls and 15 with DM) underwent repeat imaging in both the sitting and supine positions, capturing two distinct retinal images per posture. The same imaging protocol and vascular analysis software [Singapore I Vessel Assessment (SIVA), National University of Singapore, Singapore] were used for all images. Intraclass correlation coefficients were calculated to determine intra-observer reliability (Supplementary Table 1).

### Assessment of retinal vasculature

Quantitative analysis of retinal vascular parameters was performed using the semi-automated SIVA software platform. This analytical tool automatically identifies the optic disc, establishes a measurement grid referenced to the optic disc center, differentiates vessel types, and calculates comprehensive retinal vascular parameters including caliber, fractal dimension, tortuosity, and branching angles of both arterioles and venules (Fig. 2). Trained graders (YMW and TXN) verified all automated measurements and performed manual adjustments when necessary, following a standardized protocol [26]. The measurement zone was defined as the region between 0.5- and 2.0-disc diameters from the optic disc margin, with all visible vessels traversing this predefined zone included in the analysis.

### Definition of endpoints

Macula- and optic disc-centered fundus photographs were acquired at each follow-up using a nonmydriatic retinal camera (TRC 50DX, Topcon, Tokyo, Japan). Two masked graders (ZT and TXN) independently graded DR severity according to the modified Airlie House classification from the Early Treatment Diabetic Retinopathy Study [27]. This standardized 15-step severity scale categorizes retinopathy from no DR to proliferative DR. The primary endpoint for participants with NPDR or no DR at baseline was DR progression, defined as a ≥ 2-step increase on the severity scale during the follow-up period [22].

### Assessment of risk factors

The risk factors related to DR outcomes included age, duration of DM, glycated hemoglobin A1c (HbA1c), diabetic kidney disease (DKD), mean arterial blood pressure (MABP), mean ocular perfusion pressure (MOPP), body mass index (BMI) and baseline DR severity. MABP calculated as diastolic pressure plus one-third of the pulse pressure (systolic minus diastolic). MOPP was calculated by subtracting the intraocular pressure from two-thirds of the MABP. Each patient’s medical record was reviewed at each visit for the most recent fasting blood tests, including HbA1c, serum creatinine, and lipid profile [total cholesterol, high-density lipoprotein cholesterol (HDL-C), low-density lipoprotein cholesterol (LDL-C), and triglycerides]. The estimated glomerular filtration rate (eGFR) was calculated from serum creatinine based on the equation developed by the Modification of Diet in Renal Disease Study Group. DKD was defined as eGFR less than 60 mL/min/1.73 m^2^. BMI was calculated as body weight divided by body height squared. Best-corrected visual acuity was assessed at each visit using standard Snellen charts, and lens status (phakic/pseudophakic) was documented. All clinical and laboratory parameters underwent validation through the hospital’s electronic health record system before inclusion in statistical analyses.

### Statistical analysis

#### Cross-sectional analysis

Baseline characteristics and clinical characteristics were compared between healthy controls and participants with DM using the independent t-test for continuous variables and the *χ*^2^ test for categorical variables. Retinal vascular parameters were measured in sitting and supine positions for each participant. Within-group comparisons between positions were performed using the paired t-tests in the control and DM groups.

Percentage postural changes in retinal vascular parameters were calculated using the formula:

$${\text{Postural change}} = \frac{{ ( {\text{Measure at supine position}} - {\text{Measure at sitting position}} ) }}{{ {\text{Measure at sitting position}} }} \times 100\%$$

For the analysis of postural changes, participants were further stratified into three groups: healthy controls, DM without DR, and DM with DR. These postural changes were compared among the three groups using analysis of covariance (ANCOVA), adjusted for age, MABP, and DM duration. Participant was included as a random effect in all models to address inter-eye correlation. Linear trend tests were conducted to evaluate progressive changes across the three groups (from healthy controls to DM without DR to DM with DR).

#### Longitudinal analysis

Cox proportional hazards regression models estimated hazard ratios (HRs) with 95% confidence intervals (CIs) to examine the association between baseline postural changes in retinal vascular parameters and DR progression over a 5-year follow-up. All models were adjusted for the following baseline risk factors: age, HbA1c, DR severity, DM duration and MABP. A shared frailty model with a gamma distribution was incorporated to account for inter-eye correlations within participants. Schoenfeld’s global test confirmed that the proportional hazards assumption was not violated [28]. The additional predictive value of postural changes beyond currently known risk factors was assessed using the C-statistic, following the approach described by Steyerberg et al. [29] The C-statistics from multivariable models with and without the inclusion of these postural changes were then compared. Likelihood ratio tests were employed to evaluate differences in model performance.

All statistical analyses were performed using Python (version 3.11.5, Python Software Foundation). Statistical significance was defined as a two-sided *P* value <0.05.

## Results

### Patient characteristics

A total of 49 patients with DM and 38 healthy controls were enrolled in the study. The mean age was comparable between groups: 54.2 ± 13.4 years for healthy controls and 58.9 ± 11.6 years for participants with DM (*P* = 0.052). Among those with DM, the baseline DR severity included no DR (n = 23 eyes), mild NPDR (n = 6 eyes), moderate NPDR (n = 11 eyes), and severe NPDR (n = 10 eyes). The median follow-up duration for participants with DM was 59.5 months (IQR: 53.3–63.0), during which 14 eyes (28.0%) experienced DR progression. Participants with DM had higher rates of antihypertensive and lipid-lowering medication use (*P* < 0.001) and cardiovascular disease history (*P* = 0.045). Blood pressure, intraocular pressure, and ocular perfusion pressure were similar between groups. Additional demographic and clinical characteristics are presented in Table 1.

**Table 1:** Clinical characteristics of healthy controls and participants with diabetes mellitus (DM) with/without diabetic retinopathy (DR)

Characteristics	Healthy controls	Participants with DM	*P* value
Participants	n = 38	n = 49	
Age (years, SD)	54.2 (13.4)	58.9 (11.6)	0.052
Gender (male/female)	27/31	20/30	0.624
Duration of diabetes (years, SD)	–	10.64 (7.35)	–
Hemoglobin A1c (%, SD)	–	7.25 (1.12)	–
BMI (kg/m^2^, SD)	–	26.27 (3.90)	–
Total cholesterol (mmol/L, SD)	–	4.15 (0.66)	–
HDL cholesterol (mmol/L, SD)	–	1.33 (0.36)	–
LDL cholesterol (mmol/L, SD)	–	2.16 (0.55)	–
Triglycerides (mmol/L, SD)	–	1.60 (0.87)	–
Sitting systolic blood pressure (mmHg, SD)	126.8 (15.7)	130.1 (18.2)	0.320
Sitting diastolic blood pressure (mmHg, SD)	79.4 (9.2)	77.0 (10.2)	0.214
Sitting mean arterial blood pressure (mmHg, SD)	95.2 (10.4)	94.7 (11.8)	0.829
Supine systolic blood pressure (mmHg, SD)	125.7 (16.8)	131.3 (17.0)	0.091
Supine diastolic blood pressure (mmHg, SD)	75.8 (9.4)	75.1 (9.6)	0.690
Estimated glomerular filtration rate (mL/min/1.73 m^2^, SD)	–	81.4 (16.9)	–
Smoking, n (%)	2 (3.4)	4 (8.0)	0.543
Using the antihypertensive drug, n (%)	13 (22.4)	16 (69.6)	**<0.001**
Using the antihypercholesterol drug, n (%)	6 (10.3)	12 (52.2)	**<0.001**
Cardiovascular disease history, n (%)	0 (0)	5 (10.0)	**0.045**
Duration of follow-up (months), median (IQR)	–	59.5 (53.3–63.0)	–
Eyes	n = 58	n = 50	
DR severity (no DR/mild/moderated/severe NPDR), n	–	23/6/11/10	–
BCVA (logMAR, SD)	–	0.854 (0.183)	–
Lens status (phakic/pseudophakic), n	–	32/18	–
Sitting intraocular pressure (mmHg, SD)	15.1 (2.6)	15.8 (2.6)	0.221
Supine intraocular pressure (mmHg, SD)	16.8 (2.6)	17.8 (2.6)	0.053
Mean ocular perfusion pressure (mmHg, SD)	53.4 (7.0)	52.6 (7.6)	0.615
DR progression, n (%)	NA	14 (28.0)	–

*BCVA* = best-corrected visual acuity; *BMI* = body mass index; *HDL* = high-density lipoprotein; *IQR* = interquartile range; *LD*L = low-density lipoprotein; *NPDR* = non-proliferative diabetic retinopathy; *SD* = standard deviation

*P* values in bold indicate statistical significance

### Cross-sectional analysis

Table 2 compares the mean within-group changes of measurements of retinal vascular parameters of patients with DM and healthy controls when changing from the sitting to supine position. Healthy controls displayed normal physiological constriction in arteriolar caliber (92.48–89.32 μm, *P* = 0.049) and venular caliber (130.19–124.89 μm, *P* = 0.027). By contrast, those with DM and DR showed no significant change in arteriolar caliber (111.55–114.69 μm, *P* = 0.104) or venular caliber (160.84–161.89 μm, *P* = 0.880), suggesting impaired autoregulation. Notably, venular fractal dimension declined in both groups, albeit more markedly in healthy controls (1.199–1.193, *P* = 0.003) than in the DM with DR group (1.179–1.173, *P* = 0.013). Other parameters, such as arteriolar fractal dimension, tortuosity, and branching angles, showed minimal or no significant postural changes in either group.

**Table 2:** Comparison of intraocular pressure and Singapore I Vessel Assessment (SIVA) measures of retinal blood vessels between the sitting position and supine position

Variable	Healthy control (n = 58)			Participants with DM (n = 50)		
	Sitting	Supine	*P* value*	Sitting	Supine	*P* value*
	Mean ± SE	Mean ± SE		Mean ± SE	Mean ± SE	
Intraocular pressure (mmHg)	15.1 ± 0.3	16.8 ± 0.4	**<0.001**	15.8 ± 0.37	17.8 ± 0.37	**<0.001**
Retinal arteriolar caliber (µm)	92.48 ± 3.48	89.32 ± 3.00	**0.049**	111.55 ± 5.93	114.69 ± 6.17	0.104
Retinal venular caliber (µm)	130.19 ± 4.89	124.89 ± 4.40	**0.027**	160.84 ± 6.594	161.89 ± 8.28	0.880
Retinal arteriolar fractal dimension	1.199 ± 0.006	1.196 ± 0.006	0.107	1.169 ± 0.006	1.166 ± 0.007	0.169
Retinal venular fractal dimension	1.199 ± 0.006	1.193 ± 0.005	**0.003**	1.179 ± 0.006	1.173 ± 0.007	**0.013**
Retinal arteriolar simple tortuosity	1.081 ± 0.001	1.080 ± 0.001	0.162	1.081 ± 0.002	1.085 ± 0.002	0.051
Retinal venular simple tortuosity	1.091 ± 0.002	1.089 ± 0.002	0.371	1.090 ± 0.002	1.090 ± 0.002	0.773
Retinal arteriolar curvature tortuosity (×10^4^)	2.611 ± 0.132	2.720 ± 0.134	0.224	2.112 ± 0.198	2.005 ± 0.195	0.238
Retinal venular curvature tortuosity (×10^4^)	3.125 ± 0.163	3.181 ± 0.155	0.574	2.581 ± 0.261	2.432 ± 0.237	0.147
Arteriolar branching angle (degrees)	68.17 ± 1.38	68.68 ± 2.33	0.810	69.71 ± 2.90	68.61 ± 2.11	0.720
Venular branching angle (degrees)	72.58 ± 2.14	70.76 ± 2.34	0.310	72.73 ± 1.43	72.01 ± 1.77	0.828

*DM* = diabetes mellitus; *SE* = standard error

* Represents paired t-test. *P* values in bold indicate statistical significance

**Fig. 1:**
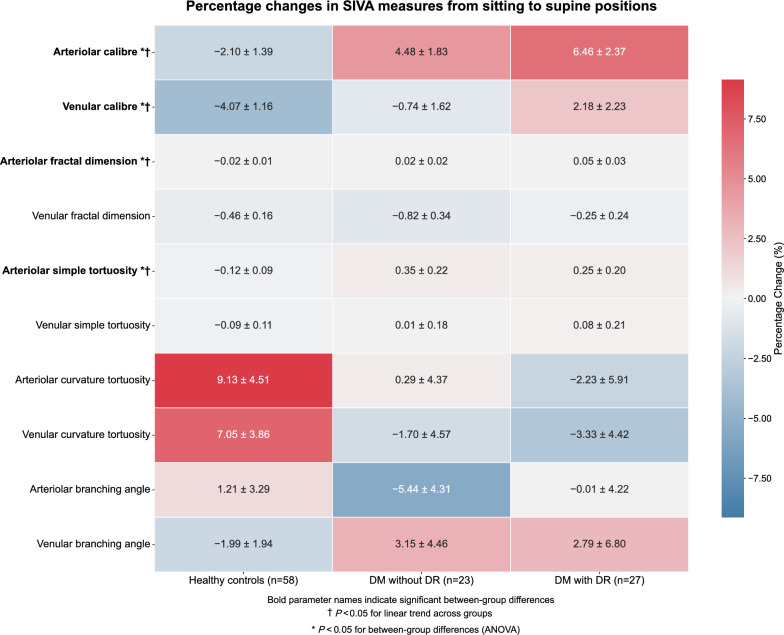
Comparisons of percentage changes in Singapore I Vessel Assessment (SIVA) measures of retinal blood vessels from the sitting to supine positions between healthy controls and diabetes mellitus (DM) patients with or without diabetic retinopathy (DR). * Adjusted for inter-eye correlation, age, mean arterial blood pressure and diabetes duration

Postural changes in retinal vascular parameters demonstrated significant differences across the three groups after adjusting for age, HbA1c, MABP, and DM duration (Fig. 1). Violin plots of the percentage sitting‑to‑supine change for key retinal vascular parameters were generated to visualize the cohort heterogeneity (Supplementary Figure 2). Retinal arteriolar caliber exhibited a strong linear trend (*P*-trend < 0.05) with healthy controls demonstrating normal constriction (−2.10% ± 1.39%), whereas participants with DM showed progressive dilation (DM without DR: +4.48% ± 1.83%; DM with DR: +6.46% ± 2.37%). Venular caliber changes also showed significant differences between groups (*P* < 0.05) and a linear trend, progressing from constriction in controls (−4.07% ± 1.16%) to mild constriction in DM without DR (−0.74% ± 1.62%) to dilation in DM with DR (+2.18% ± 2.23%). Arteriolar fractal dimension followed a gradual increase (*P*-trend < 0.05), moving from −0.02% ± 0.01% in controls to +0.02% ± 0.02% in DM without DR and +0.05% ± 0.03% in DM with DR. Meanwhile, arteriolar simple tortuosity also displayed a notable linear trend (*P*-trend < 0.05): healthy controls had a slight decrease (−0.12% ± 0.09%), whereas both DM subgroups showed increases (DM without DR: +0.35% ± 0.22%; DM with DR: +0.25% ± 0.20%). Univariable associations between systemic factors and posture‑induced retinal changes in the DM participants were also conducted (Supplementary Figure 3).

### Longitudinal analysis

Baseline percentage changes in individual retinal vascular parameter during the sitting-to-supine transition demonstrated predictive value for DR progression (Table 3). Greater retinal arteriolar tortuosity emerged as a strong risk factor, with each unit increase in percentage change corresponding to a 2.41-fold higher risk of DR progression in the multivariable model (HR = 2.41, 95% CI: 1.37–4.23; *P* = 0.002). This association strengthened from univariable analysis (HR = 1.80, 95% CI: 1.13–2.86; *P* = 0.013) to multivariable analysis, indicating independence from established clinical confounders. In contrast, wider retinal venular branching angle were associated with an estimated 45% lower risk of DR progression (HR = 0.55, 95% CI: 0.35–0.87; *P* = 0.011) in the multivariable model, improving upon the univariable result (HR = 0.65, 95% CI: 0.46–0.92; *P* = 0.013).

**Table 3:** Relationships of baseline percentage changes in Singapore I Vessel Assessment (SIVA) parameters of retinal blood vessels from sitting to supine positions to the risk of diabetic retinopathy (DR) progression

Variable	Univariable model		Multivariable model^a^	
	HR (95% CI)	*P* value	HR (95% CI)	*P* value
Arteriolar caliber	0.76 (0.51–1.13)	0.169	0.81 (0.51–1.29)	0.390
Venular caliber	0.81 (0.48–1.36)	0.428	0.84 (0.49–1.44)	0.532
Arteriolar fractal dimension	0.98 (0.61–1.57)	0.930	1.02 (0.48–2.17)	0.962
Venular fractal dimension	0.71 (0.42–1.20)	0.202	0.76 (0.36–1.60)	0.462
Arteriolar simple tortuosity	**1.80 (1.13–2.86)**	**0.013**	**2.41 (1.37–4.23)**	**0.002**
Venular simple tortuosity	1.10 (0.61–1.99)	0.743	1.16 (0.66–2.04)	0.603
Arteriolar curvature tortuosity	1.11 (0.42–2.91)	0.838	1.14 (0.38–3.41)	0.813
Venular curvature tortuosity	0.84 (0.39–1.81)	0.740	0.86 (0.33–2.24)	0.762
Arteriolar branching angle	0.82 (0.31–2.15)	0.683	0.75 (0.22–2.57)	0.650
Venular branching angle	**0.65 (0.46–0.92)**	**0.013**	**0.55 (0.35–0.87)**	**0.011**

*CI* = confidence interval

Bold text indicates statistically significant associations with DR progression (*P* < 0.05)

^a^Adjusted for inter-eye correlation, age, duration of diabetes, glycated hemoglobin, diabetic retinopathy severity, mean arterial blood pressure at baseline

**Fig. 2:**
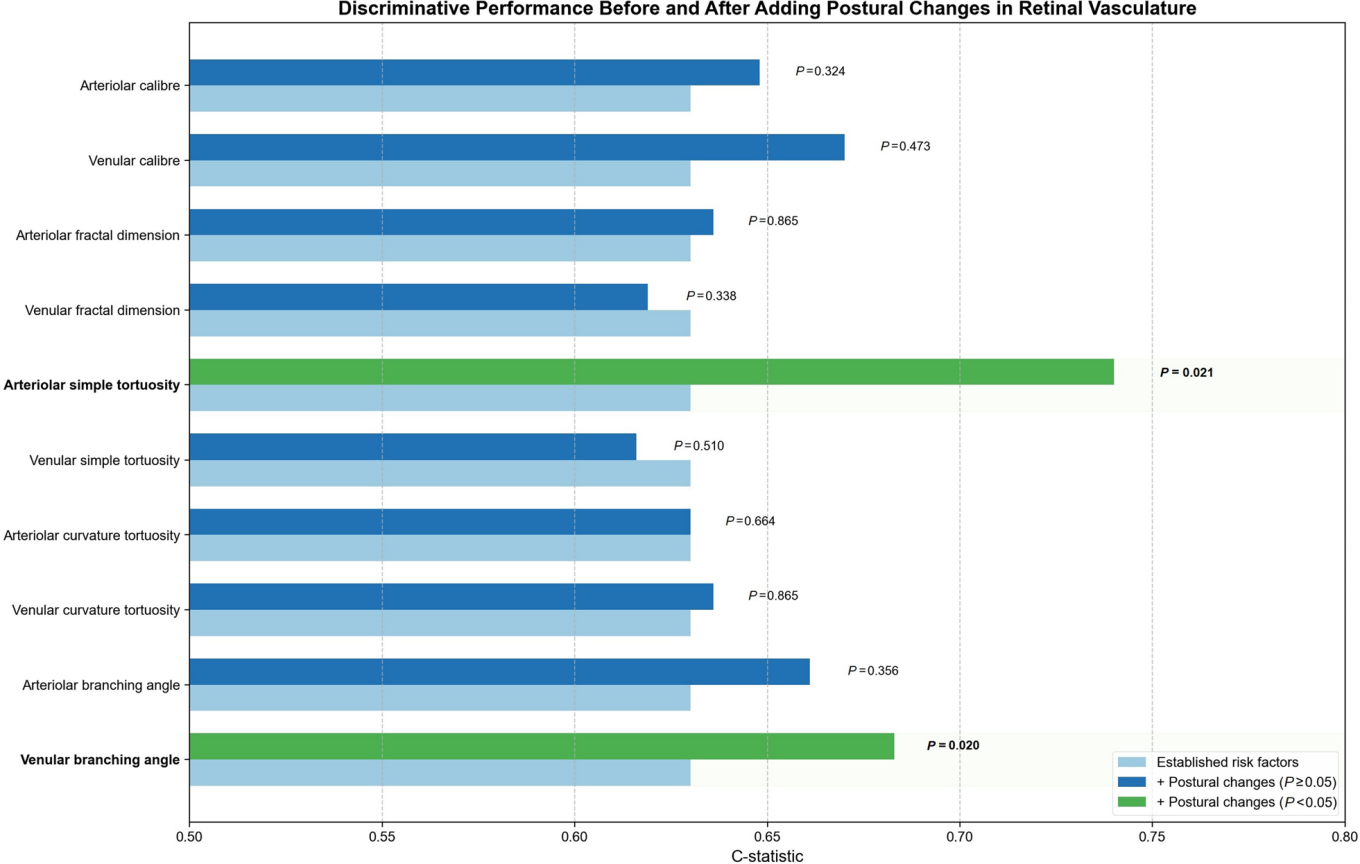
The enhancement of discriminative performance after including postural changes in retinal vasculatures in the multivariable model. Established risk factors including age, duration of diabetes, glycated hemoglobin, mean arterial blood pressure, diabetic retinopathy severity at baseline. *P* values calculated using Likehood Ratio Test. Bold *P* values indicate statistical significance (*P* < 0.05)

To assess incremental discriminative performance, these postural changes were added to a base model containing established risk factors (Fig. 2). Arteriolar simple tortuosity improved the model’s discrimination (C-statistic increasing from 0.630 to 0.740; *P* = 0.021), while venular branching angle raised it from 0.630 to 0.683 (*P* = 0.020). Both enhancements were statistically significant according to likelihood ratio tests. No other retinal vascular parameters offered notable improvements in discriminative performance (all *P* > 0.05). Since follow-up frequency was strictly determined by baseline DR severity, we performed a sensitivity analysis using Cox regression stratified by baseline DR grade. This approach effectively controls for differential surveillance schedules by analyzing progression risk within each severity stratum separately, thereby eliminating the potential confounding effect of varying follow-up intervals (Supplementary Table 2).

## Discussion

This pilot study demonstrated that postural changes in retinal vascular parameters provide significant prognostic information for DR beyond currently known risk factors. Using smartphone-based retinal imaging, we found marked differences in sitting-to-supine vascular responses among healthy controls, type 2 DM patients without DR, and those with established DR. These dynamic responses emerged as potentially valuable indicators of microvascular dysfunction. Most importantly, longitudinal analyses of this study revealed that specific postural changes in retinal vascular geometry, particularly greater arteriolar tortuosity and wider venular branching angles, independently predicted DR progression. These findings suggest that quantifying vascular response to postural changes may enhance risk stratification, potentially enabling earlier targeted intervention in high-risk individuals with type 2 diabetes.

Cross-sectional analyses of this study revealed marked differences in postural-induced retinal vascular responses among healthy controls, participants with DM without DR, and those with DR. In healthy individuals, we observed significant reductions in arteriolar and venular calibers upon transitioning to the supine position. The magnitude and direction of this constriction align with the normal retinal myogenic response described in prior physiological studies, which serves to maintain stable blood flow against increased perfusion pressure [30, 31]. By contrast, participants with DM showed diminished or entirely absent caliber changes. This distinct deviation from the expected healthy response, characterized by a shift from protective constriction to paradoxical dilation, indicates valid vascular dysregulation consistent with endothelial dysfunction, rather than measurement variability [14, 31]. This pattern extended to other retinal vascular parameters. While healthy controls demonstrated relatively consistent or adaptive shifts in fractal dimension, branching angle, and tortuosity, those with DM exhibited less pronounced or even paradoxical postural changes in these metrics. These discrepancies imply a hierarchy of vulnerability within the retinal microvasculature, wherein caliber responsiveness appearing most susceptible, potentially foreshadowing future structural remodeling [2, 10]. Notably, arteriolar tortuosity showed a stepwise increase from healthy controls to those with DM, particularly pronounced in those with DR, suggesting ongoing vascular remodeling driven by chronic hyperglycemia. While we did not perform specific autonomic function tests (e.g., heart rate variability or tilt-table testing) to assess the associations with other clinical manifestations of diabetic autonomic neuropathy, we did not observe overt orthostatic hypotension in our cohort after measuring blood pressure in both sitting and supine positions. The “defective myogenic response” in the retina likely mirrors systemic autonomic neuropathy and endothelial dysfunction. Future studies are warranted to correlate these retinal findings with systemic autonomic markers.

Longitudinal analyses of this study revealed that postural changes in two key retinal vascular parameters significantly enhance the prediction of DR progression. First, greater arteriolar tortuosity emerged as a robust, independent risk factor for DR progression (HR = 2.41, 95% CI: 1.37–4.23; *P* = 0.002). This exaggerated tortuosity response likely reflects endothelial dysfunction from chronic hyperglycemia, which reduces nitric oxide bioavailability and impairs autoregulatory vasoconstriction [24, 32, 33]. When confronted with postural fluctuations in perfusion pressure, arterioles with compromised wall integrity fail to maintain a stable vessel configuration, particularly its tortuosity. Second, wider venular branching angles were strongly associated with a lower risk of DR progression (HR = 0.55, 95% CI: 0.35–0.87; *P* = 0.011), possibly indicating preserved venular compliance that remain unaffected by basement membrane thickening, pericyte loss, and decreased elasticity [34, 35]. The ability to dynamically adjust branching architecture in response to postural changes may help mitigate pathological flow disturbances and resultant tissue hypoxia [36]. These findings suggest that postural changes in retinal vascular geometry reflect underlying endothelial dysfunction and impaired autoregulation of retinal blood flow associated with diabetes in daily life.

The clinical implications of these findings are considerable. Dynamic retinal vascular imaging using smartphone-based photography could offer a cost-effective method to enhance DR screening and risk stratification, particularly in primary care or resource-limited settings [37]. Our results show that retinal vascular geometric variables significantly improve DR risk prediction beyond currently established clinical risk factors. By identifying impaired vascular responsiveness before clinical DR onset, clinicians can implement targeted interventions and personalized monitoring strategies. Patients showing abnormal vascular responses might benefit from more frequent monitoring or earlier management of modifiable risk factors. This approach shifts DR management from reactive to proactive. Furthermore, smartphone-acquired fundus images can be easily integrated with telemedicine platforms and combined with artificial intelligence-powered automated image analysis. Leveraging such automated capabilities directly on smartphones could facilitate risk assessment, enhance accessibility to retinal screening, and support non-specialists in remote settings. Integration with electronic records and telehealth platforms would enable sophisticated risk prediction models, while user-friendly adapters and automated tools could expand access to non-specialist settings.

This study has several strengths. While previous studies have documented associations between static parameters (such as vessel caliber and tortuosity) and DR risk [21, 24], our study explored dynamic changes in these parameters that occur in daily life and their predictive value for DR progression. Additionally, our approach combining smartphone-based imaging demonstrates the feasibility of this approach for potential wide-scale implementation, particularly in primary care or resource-limited settings. Despite these promising results, several limitations need to be considered. First, a formal sample size calculation was not performed as this study was designed as an exploratory pilot study to generate preliminary evidence on a novel methodology. Although the high rate of DR progression in our cohort (28%) facilitated the detection of significant associations, the overall sample size was modest. The relatively modest sample size (n = 49) and limited progression events (n = 14) restrict statistical power and generalizability to detect smaller yet clinically relevant effects, and these findings should be interpreted as hypothesis-generating and require validation in larger, multi-center cohorts. Second, the number of participants who developed DR (transitioning from no DR at baseline) was relatively low, limiting the capacity to investigate the relationship between postural changes in retinal microvasculature and incident DR. Third, potential unmeasured confounders, such as genetic predispositions or environmental factors, could also influence retinal vascular changes and DR risk [38]. Additionally, detailed categorization of glucose-lowering medication classes, specifically GLP-1 receptor agonists and SGLT2 inhibitors which may have direct vasoactive effects, was not available for analysis in this cohort. Future studies are essential to validate the findings of this study and expand our understanding of how dynamic retinal vascular assessments can optimally inform DR screening and management.

## Conclusions

In conclusion, postural changes in retinal vascular parameters, particularly arteriolar tortuosity, provide additional prognostic information for DR progression beyond currently known clinical risk factors. Smartphone-based imaging offers a practical and cost-effective method to detect subclinical microvascular dysfunction at earlier stages, allowing more precise risk stratification and timely interventions.

## Supplementary Information

Additional file 1.

## Data Availability

The datasets used and/or analyzed during the current study are available from the corresponding author on reasonable request.
