# Supplementary material for: Postural changes in retinal vascular parameters and risk of diabetic retinopathy progression in type 2 diabetes mellitus: a pilot study
Source: Eye Vis (Lond). 2026 Jan 6;13:3. doi: 10.1186/s40662-025-00471-z (PMC12772113; doi:10.1186/s40662-025-00471-z)
Supplement: Supplementary file 1 — Additional file 1. [file 40662_2025_471_MOESM1_ESM.docx]

**Supplementary Figure 1**. Smartphone fundus imaging in sitting and supine positions with SIVA vascular quantification. **a** Imaging postural changes in retinal vasculature using a smartphone with a novel clip-on adapter lens. **b** Using Singapore I Vessel Assessment (SIVA) software to measure a spectrum of retinal vascular parameters (caliber, fractal dimension, tortuosity and branching angle) from a smartphone retinal image imaged from a subject with diabetes mellitus (DM).


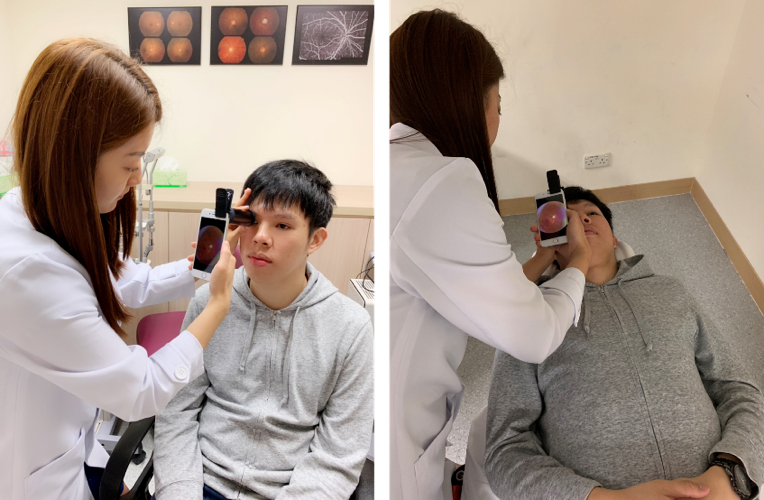


**a**

**b**


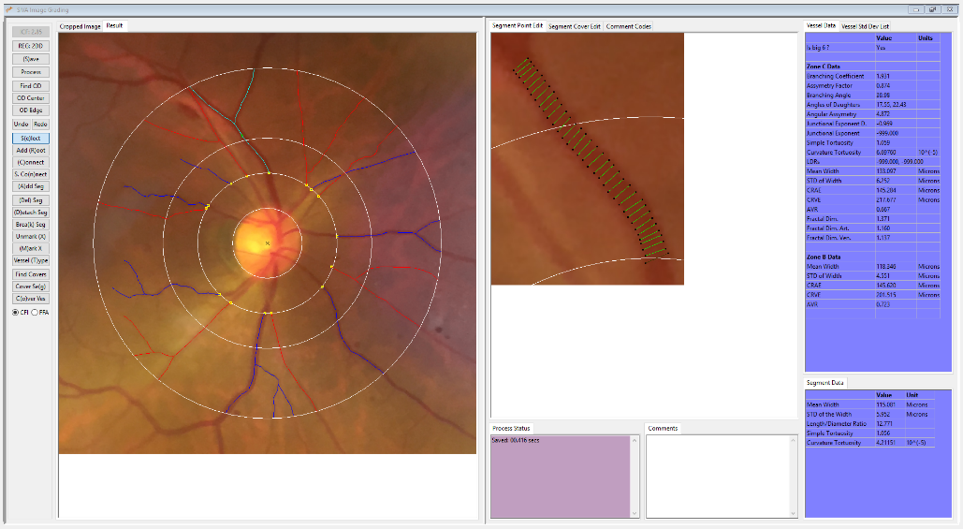


**Supplementary Figure 2**. Violin plots of the distribution of postural changes in retinal vascular parameters stratified by disease severity and progression status. The distribution of percentage change from the sitting to supine position for key retinal vascular parameters are shown. The width of the violin represents the probability density of the data, while individual data points are overlaid to visualize cohort heterogeneity. The gray dashed line indicates no change (0%).


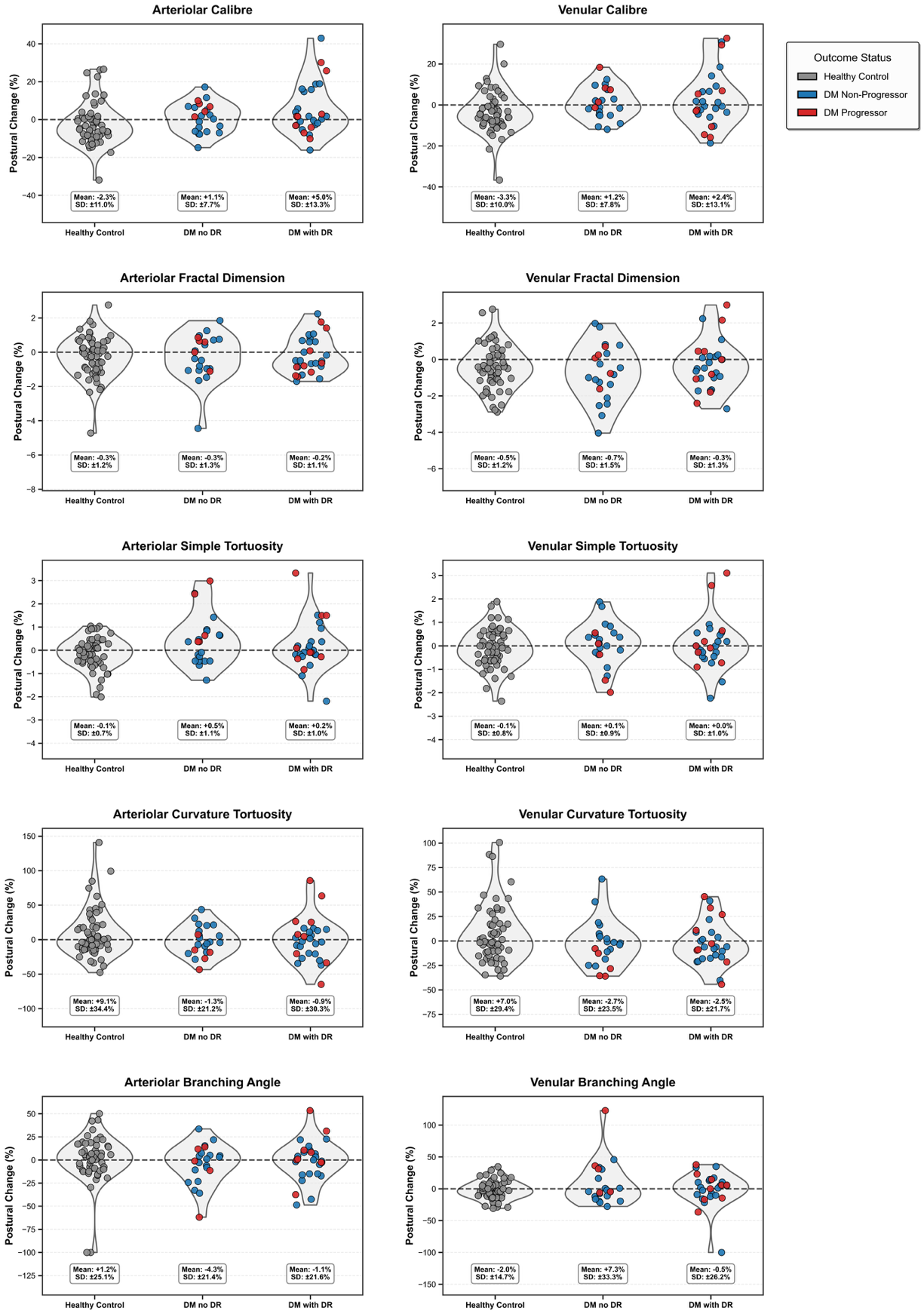


**Supplementary Figure 3.** Univariate associations between systemic factors and posture‑induced retinal changes in the diabetes mellitus (DM) participants.


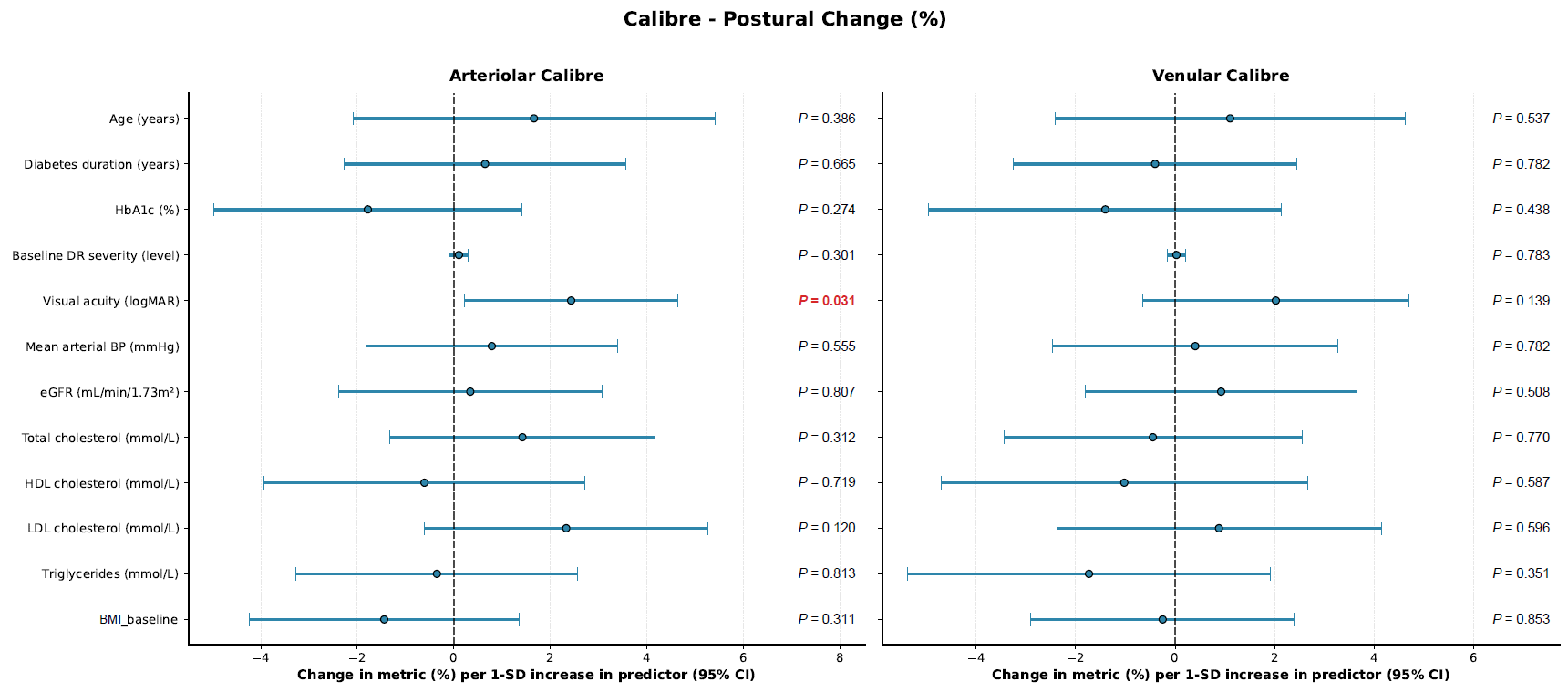


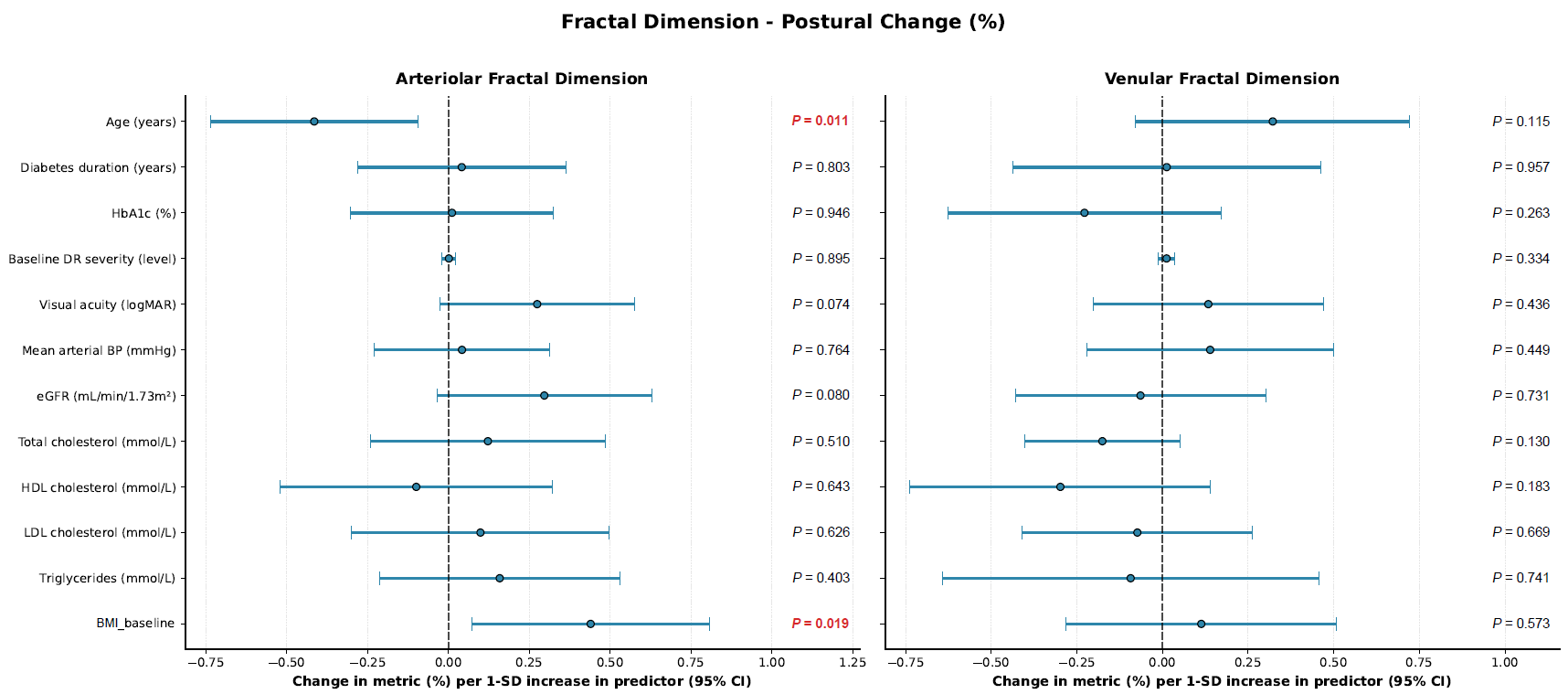


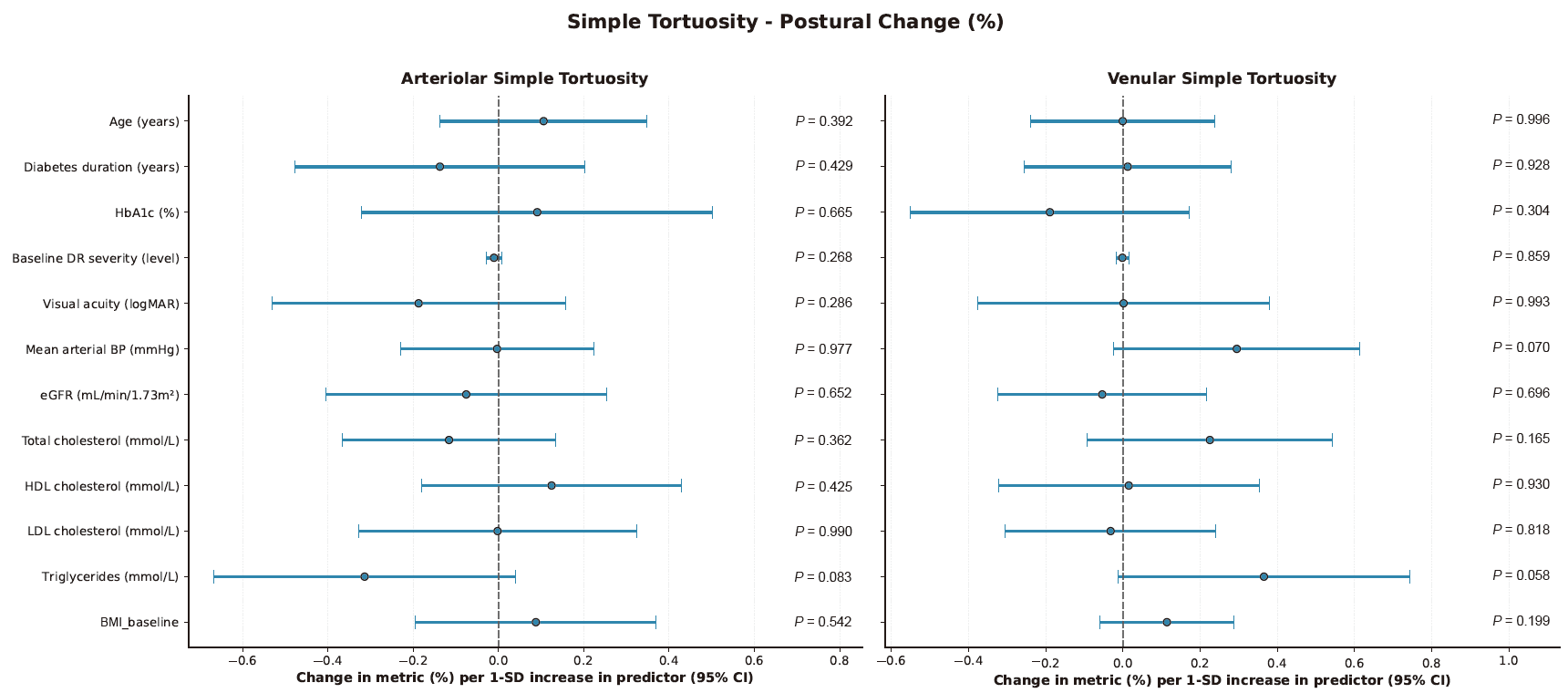


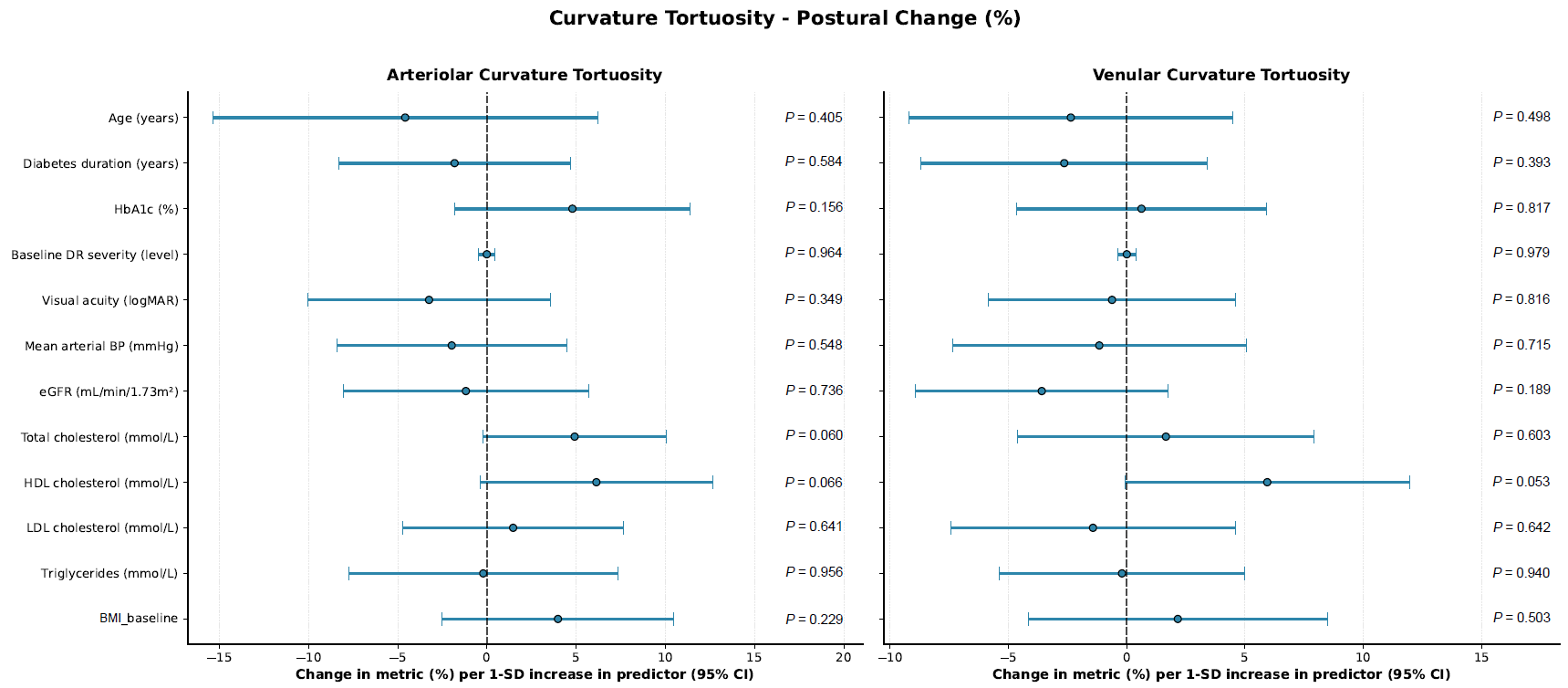


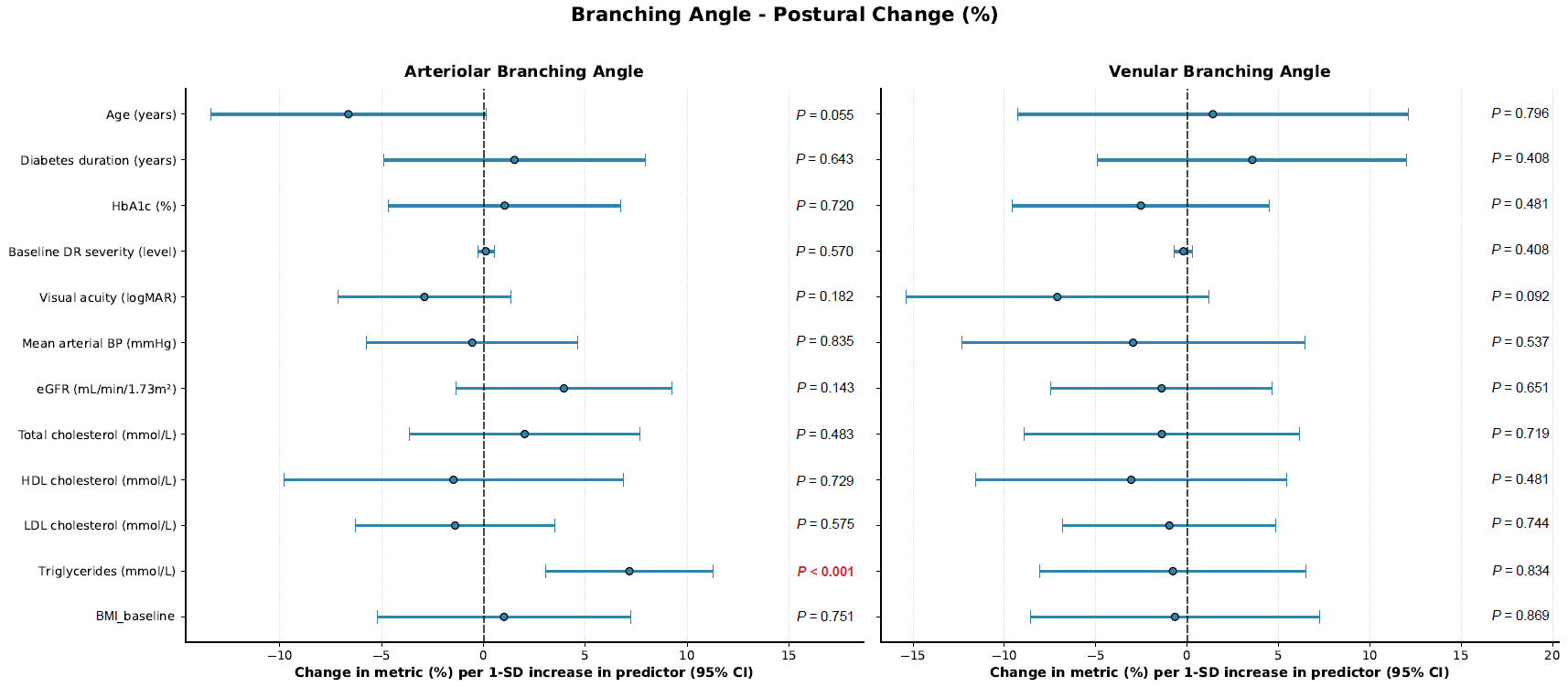


**Supplementary Table 1.** Reliability of Singapore I Vessel Assessment (SIVA) measures from smartphone retinal images in a subset of 30 participants (15 controls + 15 subjects with DM).

| **Variable** | **Sitting position** | |  | **Supine position** | |
| --- | --- | --- | --- | --- | --- |
|  | **ICC** | **95% CI** |  | **ICC** | **95% CI** |
| Arteriolar caliber | 0.996 | (0.996 to 0.999) |  | 0.998 | (0.995 to 0.999) |
| Venular caliber | 0.997 | (0.994 to 0.999) |  | 0.997 | (0.994 to 0.999) |
| Arteriolar fractal dimension | 0.951 | (0.896 to 0.977) |  | 0.912 | (0.818 to 0.958) |
| Venular fractal dimension | 0.968 | (0.932 to 0.985) |  | 0.950 | (0.895 to 0.977) |
| Arteriolar simple tortuosity | 0.830 | (0.665 to 0.917) |  | 0.876 | (0.751 to 0.941) |
| Venular simple tortuosity | 0.792 | (0.535 to 0.898) |  | 0.904 | (0.803 to 0.954) |
| Arteriolar branching angle | 0.796 | (0.601 to 0.902) |  | 0.814 | (0.628 to 0.912) |
| Venular branching angle | 0.872 | (0.743 to 0.939) |  | 0.854 | (0.710 to 0.930) |
| Arteriolar branching coefficient | 0.814 | (0.631 to 0.911) |  | 0.827 | (0.651 to 0.918) |
| Venular branching coefficient | 0.858 | (0.717 to 0.932) |  | 0.842 | (0.688 to 0.924) |

ICC = intraclass correlation coefficient; CI = confidence interval

**Supplementary Table 2.** Sensitivity Cox regression for posture‑induced retinal metrics and risk of diabetic retinopathy (DR) progression.

| **Metric (per 1‑SD)** | **Model specification** | **HR (95% CI)** | ***P* value** |
| --- | --- | --- | --- |
| Arteriolar simple tortuosity %Δ | Primary stratified model | 2.38 (1.36–4.18) | 0.002 |
|  | + Mean arterial BP | 2.40 (1.35–4.24) | 0.003 |
|  | + eGFR | 2.53 (1.31–4.90) | 0.006 |
|  | + Triglycerides | 2.08 (1.17–3.70) | 0.012 |
| Venular branching angle %Δ | Primary stratified model | 0.55 (0.36–0.82) | 0.003 |
|  | + Mean arterial BP | 0.54 (0.35–0.85) | 0.008 |
|  | + eGFR | 0.54 (0.36–0.83) | 0.004 |
|  | + Triglycerides | 0.53 (0.33–0.88) | 0.014 |

HR = hazard ratios; CI = confidence interval; BP = blood pressure; eGFR = estimated glomerular filtration rate

Models stratified by baseline DR grade to control for differential follow-up schedules. All models adjusted for age, diabetes duration, and HbA1c.
